# Limits and mechanisms of honey bee colonial thermoregulation in the heat

**DOI:** 10.1242/jeb.252357

**Published:** 2026-07-23

**Authors:** Jon F. Harrison, Jun Chen, Jordan R. Glass, Cahit Ozturk, Brian H. Smith, Jennifer H. Fewell, Gloria DeGrandi-Hoffman, Yun Kang, Brittany Sorensen, David Rothweil, Nicole S. DesJardins, Adrian Fisher

**Affiliations:** ^1^ School of Life Sciences, Arizona State University, Tempe, AZ 85287-4501, USA; ^2^ Department of Mathematics, Texas A&M University – Kingsville, Kingsville, TX 78363, USA; ^3^ College of Integrative Science and Arts, School of Applied Sciences and Arts, Arizona State University, Mesa, AZ 85212-2780, USA; ^4^ Science and Mathematics Faculty, College of Integrative Science and Arts, Arizona State University, Mesa, AZ 85212-2780, USA

**Keywords:** *Apis mellifera*, Heat stress, Heat budget, Pollination, Thermal limits, Climatic warming, Social insect

## Abstract

Pollinators play critical roles in food sustainability and terrestrial ecosystems, yet we lack understanding of the limits and mechanisms of colonial thermoregulation during heat stress. Despite the extensive behavioral studies of thermoregulation in honey bees and the recognition that temperature extremes impact colony growth and survival, the upper limits of honey bee colony thermoregulation and survival under extreme heat have not been well studied. Textbooks and reviews state that honey bees can maintain internal conditions at air temperatures up to 60°C. Here, we provide the first measures of colonial gas exchange for standard honey bee colonies during an extreme but realistic heat stress, and provide the first quantitative assessment of the thermoregulatory capacities of a heat-stressed *Apis mellifera* hives. Air temperatures of 50°C for 4 h caused brood areas to reach damaging 38°C in most colonies. Heat stress reduced pollen foraging, induced fanning and bearding behavior, suppressed colonial metabolic rate and elevated evaporative water loss. Contrary to textbook paradigm, increased evaporative water loss was not the primary mechanism of colonial thermoregulation in the heat. Rates of heat buffering mechanisms during this heat challenge were passive heat storage in honey and wax (76%) and in bees (14%), metabolic suppression (7%) and increased evaporative heat loss (3%). Hive temperatures fall at night, allowing passive buffering to reduce diurnal heat stress. Social regulation of behavior, physiology and nest traits provide heat mitigation, but projected heat waves may exceed these capacities.

## Teaser

**Highlighted Article:** Protection of honey bee colonies from heat injury depends on passive heat buffering of honey and wax, with contributions from suppression of metabolic heat production and increased evaporative heat loss.

## INTRODUCTION

As the planet warms, heat waves are predicted to cause mass mortality events for organisms, and to be of more concern than rises in mean temperatures (Johnson et al., 2023; Kingsolver et al., 2021; Marx et al., 2021; Meehl and Tebaldi, 2004; Parmesan et al., 2000). Pollinators are critical for both agriculture and the function of natural terrestrial communities; therefore, understanding their susceptibility to heat waves is necessary to predict the consequences of environmental warming on food and ecosystem sustainability (Gallai et al., 2009; Goulson, 2022; Hanley et al., 2015; Harvey et al., 2020, 2023; Wagner et al., 2021). The nests of pollinators may be particularly vulnerable to climate change as nests are usually stationary and represent significant energy investments (Johnson et al., 2023; Jones and Oldroyd, 2007). Highly social pollinators such as honey bees, however, possess sophisticated behavioral and physiological mechanisms to thermoregulate their nests (Johnson et al., 2023; Jones and Oldroyd, 2007; Simpson, 1961; Stabentheiner et al., 2021). Although the behavioral responses to heat stress have been well demonstrated for honey bee colonies, we lack a quantitative, mechanistic description of the relative contributions of different strategies to overall colonial thermoregulation for honey bees or any social insect colony.

Mechanisms by which honey bee colonies cope with heat stress include behaviorally controlled evaporative cooling, metabolic heat suppression and passive heat absorption, but these have not previously been quantified for intact, functioning hives (Jones and Oldroyd, 2007; Simpson, 1961; Stabentheiner et al., 2021). The behavioral regulation of evaporative cooling has been best studied. When brood temperatures rise, nurses solicit water from nestmates, inducing water collection by the foragers (Kühnholz and Seeley, 1997; Ostwald et al., 2016). Workers spread water on the brood, while nestmates fan with their wings to increase air circulation through the hive, producing evaporative cooling (Chadwick, 1931; Hazelhoff, 1954; Lindauer, 1954; Southwick and Moritz, 1987; Stabentheiner et al., 2021). Workers can also form insulating heat shields to absorb heat with their bodies (Bonoan et al., 2014; Starks and Gilley, 1999), and to reduce heat production and disperse heat away from the brood during periods of heat stress (Jhawar et al., 2023; Stabentheiner et al., 2021). Although it is well known that clusters and frames of bees can reduce oxygen consumption as temperature rises (Kronenberg and Heller, 1982; Southwick, 1985), no studies have measured gas exchange of full-scale, functioning colonies, or examined whether this extends to the thermal ranges where colonial heat stress occurs.

Worker behaviors can regulate nest temperatures, but nest characteristics and structure also can play important roles in thermoregulation (Jones and Oldroyd, 2007). Biophysical models demonstrate that the thermal mass of honey and wax is large and should strongly influence the time course of hive warming in response to a heat stress (Lu et al., 2025; Mitchell, 2016). The bees themselves also have considerable mass within a colony and may also serve as a passive heat store in response to warming or cooling, if hive and bee body temperatures vary with the hive temperature (Stabentheiner et al., 2021). To date, no studies are available that have quantitatively evaluated the relative importance of increased evaporative cooling, metabolic suppression and passive heat storage when honey bee colonies are challenged with realistic heat wave conditions.

Similarly, the limits of heat tolerance for honey bee colonies, and, indeed, social insect colonies generally, are poorly understood (Johnson et al., 2023). Honey bee brood require strict thermoregulation at 34–36°C for optimal development to occur (Groh et al., 2004; Himmer, 1927). If pupae are held at 37°C or 38°C for their entire development, pupal survival decreases to 40% and 0%, respectively (Groh et al., 2004). Even short-term heat exposures have negative consequences; pupal exposure to 40°C for 1 h on six consecutive days increases forewing shape asymmetry and reduces lifespan in honey bee workers (Medina et al., 2018). Additionally, the viability of stored sperm in honey bee queens decreases if their body temperatures rise above 38°C for more than a few hours (McAfee et al., 2020).

**Table:** 

List of symbols and abbreviations	
*B*	Passive heat storage in bees (J)
*F* e_CO_2__	Fractional content of carbon dioxide in the excurrent air from the respiratory mask
*F* e_H_2_O_	Fractional content of water in the excurrent air from the respiratory mask
*F* e_O_2__	Fractional content of oxygen in excurrent air from the respiratory mask
*F* i_CO_2__	Fractional content of carbon dioxide in the air flowing into the respiratory mask
*F* i_H_2_O_	Fractional content of water in the air flowing into the respiratory mask
*F* i_O_2__	Fractional content of oxygen in the air flowing into the respiratory mask
FR_i_	Flow rate (STP ml min^−1^) into the respiratory mask
*H*	Rate of heat storage in honey (W)
SH_B_	Specific heat of honey bees (J g^−1^ °C^−1^)
*V*_CO_2__	Carbon dioxide emission rate of the colony (STP ml min^−1^)
*V*_H_2_O_	Rate of water loss of the colony (STP ml min^−1^)
*V*_O_2_ _	Oxygen consumption rate of the colony (STP ml min^−1^)
Δ*T*_B_	Rise in temperature of a bee during heat stress (°C)
Δ*T*_be_	Rise in temperature of brood edge during heat stress (°C)
Δ*T*_p_	Rise in temperature of hive periphery during heat stress (°C)

Owing to the negative impacts of temperatures just a few degrees above optimal on brood development and reproduction, a key question is how well colonies can thermoregulate during realistic heat stresses. A single honey bee colony was reported to maintain brood temperatures at 36°C at ambient temperatures of 60°C (Lindauer, 1955), and Bordier et al. (2017) found that exposing honey bee colonies for 5 days to an air temperature of 37°C did not affect temperatures in brood area, suggesting that honey bee colonies can successfully thermoregulate during such heat waves. Although 37–40°C can be a realistic heat wave in some regions, maximal air temperatures near or above 50°C have been observed recently during heat waves in Africa, Asia, North America and Australia (Perkins-Kirkpatrick et al., 2024). Furthermore, climate models predict that air temperatures over 50°C will become increasingly common in many low-latitude parts of the world over the next century (Donat et al., 2020; Kornhuber et al., 2024). Thus, a critical question for beekeepers, food sustainability and ecological function is whether colonies will be able to maintain brood temperatures in the optimal range when maximum ambient air temperatures exceed 50°C.

To provide a context for our heat stress test, we examined natural diurnal variation in hive temperatures to evaluate the potential role of nocturnal cooling in passive amelioration of diurnal heat stress. For the heat stress test, we experimentally exposed honey bee colonies to air temperatures of approximately 50°C for a median duration of 4.5 h. During this heat stress test, we tested the colony's ability to regulate temperatures at the brood center, brood edge and hive periphery. We stopped the experiment when brood temperatures reached 38°C, a temperature known to be lethal to honey bee brood if sustained (Groh et al., 2004). To assess thermoregulatory mechanisms, we provide the first measurements of gas exchange (oxygen, carbon dioxide and water) for intact, whole honey bee colonies. We tested the hypothesis that honey bee colonies suppress metabolic heat production and elevate evaporative heat loss in response to heat stress. We also quantitatively compared the heat mitigation contributions of these physiological responses with that of passive heat storage in bees, honey and wax.

## MATERIALS AND METHODS

### Diurnal thermal variation within an undisturbed honey bee colony

Recently, we assessed diurnal and seasonal changes in internal colonial temperatures for honey bees during a hot desert summer at Arizona State University's apiary. That study found considerable diurnal thermal variation, including in the brood center and edge, with the magnitude of diurnal variation increasing on hotter days and in smaller colonies (Chen et al., 2026). For comparison with the data in this study, we examined such diurnal variation for a single hive with very similar adult population and honey stores as our average colony, on one typical summer day. Methods are described in detail in Chen et al. (2026), but briefly, temperatures of the brood center, brood edge and hive periphery in each hive box were monitored every 30 min using Telid^®^ RFID temperature loggers (Microsensys, Erfurt, Germany). The number of adults and honey stores were measured as described below.

### Experimental design for heat stress test

**Fig. 1.**
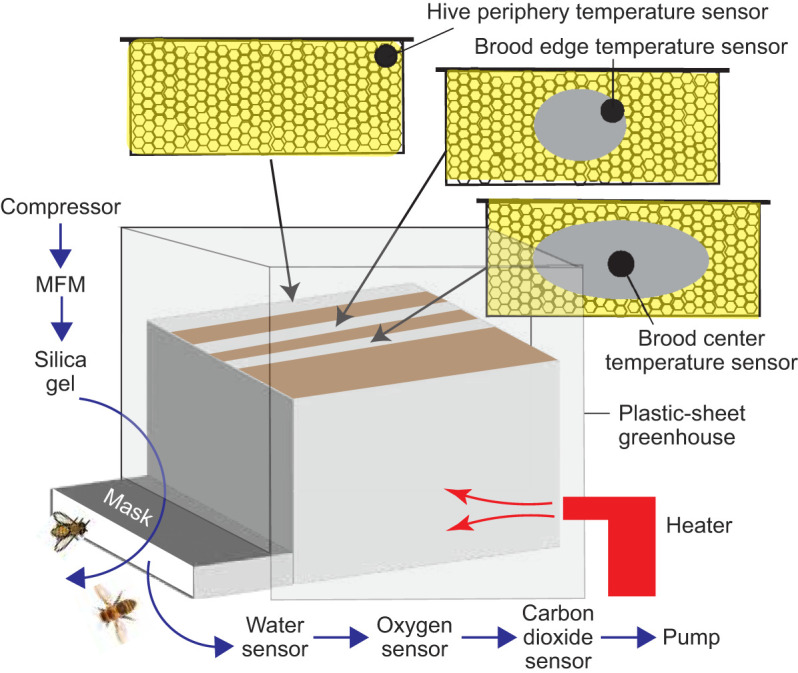
**Setup for studying the effect of heat stress on thermoregulation and gas exchange of a honey bee colony.** Single hive boxes contain 10 frames; three of these are extracted from the box shown here. Brood (eggs, larvae, pupae) are mostly located in an ellipsoid volume centered on one of the inner-most frames, usually extending over 3–4 frames. Grey ellipses indicate areas with brood; yellow indicates regions with mostly honey and pollen. Temperature sensors (round black circles) were mounted near on the periphery on one of the outermost frames, at the edge of the brood and near the center of the brood. A respiratory mask on the front of the hive allowed bees to enter and leave, and was flushed at a known rate by partially dried air flow measured by a mass flow meter (MFM). Air was drawn from the mask by a pump, through the gas analyzers. During the experiment, the three frames are within the hive box, covered with bees. Not shown is the hive box lid, which was taped tightly to the hive box to prevent air leaks. Tape was also used to prevent leaks where the respiratory mask inserted into the hive box. A plastic-sheeted greenhouse encloses the hive box (but not the compressor, MFM or gas sensors), through which the mask extends so bees can forage. An electric heater outside the greenhouse forced hot air into the greenhouse. Some colonies had two stacked hive boxes, with additional temperature sensors.

Our goal was to quantitatively measure the mechanisms by which honey bee colonies thermoregulate in response to heat, and test whether exposure to air temperatures of >50°C cause brood temperatures to exceed those known to be damaging in the long term (38°C; Groh et al., 2004). Each colony was heat-stress tested once, with eight colonies tested in the summer of 2022 and seven during the summer of 2024. On the day of the heat-stress test, early in the morning, temperature loggers were placed on top of the hive (to measure air temperature in the plastic box enclosing the hive) and in the hive, with a single logger placed at each of the following locations: the hive entrance, the three-dimensional center of the brood within the hive, the edge of the brood area, on a frame at the outer edge of the lower hive box (designated hive periphery) and on a frame at the outer edge of the top hive box (if the hive had two boxes; Fig. 1). The respiratory mask (described below) was inserted into the hive entrance; this mask was flushed with ambient air generated by an air compressor and then subsampled with airflow to oxygen, carbon dioxide and water sensors to measure colonial gas exchange (Fig. 1). The mask was open to air, so bees could continue to forage. The hive was covered with a cubic plastic enclosure (1.09 m sides, 1.30 m^3^ volume) that was taped around the mask entrance, so the hive was enclosed in an air space that could be heated while bees were free to forage. We used standard Langstroth hives (0.41×0.51 m, width×length, with either one or two 0.24 m high boxes). Before any heating occurred (07:00–09:30 h), but after the mask and enclosure were placed on the hive, we measured the number of fanners, pollen foragers and non-pollen foragers, temperatures of all loggers, and colonial gas exchange. At this time, air temperatures varied from 23°C to 41°C, averaging 29°C. Total time from placing the enclosure over the hive, including all behavioral and physiological measurements to the initiation of heating, was 30–60 min. Then the hive was heated by an electric heater (1500 W, GiveBest^®^, PTC-905) with a fan that pushed hot air into the enclosure, while we monitored enclosure and hive temperatures (Fig. 1). When brood center temperatures reached 38°C (median duration of heating=4.5 h, range 2–7 h), we stopped the heating to protect the hive, and again measured the number of fanners, pollen foragers and non-pollen foragers, temperatures of all loggers, bearding behavior and colonial gas exchange.

### Animals

Colonies of Italian honey bees (*Apis mellifera ligustica* Spinola 1806) were established in April 2022 and 2024 using frames and workers obtained from the Arizona State University (ASU) apiary; colony characteristics are shown in Table 1. All colonies were requeened with queens from Pendall Apiaries (Stonyford, CA, USA) in April 2022 and again in April 2024. The apiary is close to both urban gardens and farms, so forage was readily available during the experiments, though food availability was not controlled. Colonies were positioned in a row, parallel to the north wall of the main apiary building on the ASU Polytechnic campus honey bee laboratory in Mesa, AZ, USA (33.293172, −111.684520). Colonies were positioned approximately 0.75 m from the building and 1 m apart, and experienced partial sun exposure. Multiple water sources were available within 100 m of the hives, including a large artificial pond dotted with rocks for bees to stand on while drinking. At this location, average summer temperatures (June to August) can vary from 23°C to 40°C, and maximum shaded air temperatures can exceed 45°C (Chen et al., 2026).

**Table 1.** Characteristics of experimental hives

Hive no.	No. boxes	No. adult workers	Capped honey (cm^2^)	Uncapped honey (cm^2^)	Pollen (cm^2^)
1	2	16,417	NM	NM	NM
3	2	13,690	NM	NM	NM
5	1	3374	NM	NM	NM
6	2	13,408	NM	NM	NM
7	2	24,551	NM	NM	NM
8	1	4755	NM	NM	NM
9	2	18,419	NM	NM	NM
10	2	25,974	NM	NM	NM
14	2	18,804	15,268	6479	903
19	2	24,530	11,976	8855	398
24	2	12,960	8028	12,289	1023
25	2	20,881	13,229	4587	2784
26	1	6466	3855	5058	13.7
27	2	11,561	7624	1877	1256
28	2	18,644	4614	3188	1285

Honey and pollen were measured only in 2024 (NM, not measured).

### Temperature loggers

The temperature loggers were Microsensys TELID710-Q3088 temperature beacons (circular, 27 mm diameter, 13 mm thick, 0.1°C resolution). These were pushed into the wax at the appropriate location (Fig. 1). The loggers communicate with an Android smartphone using Bluetooth, enabling us to track the temperatures inside the hive as needed.

### Behavior: foraging, fanning and bearding

Foraging behavior was assessed before any heating and at the end of the heat-stress test by visually observing the hive entrance and counting the number of returning bees (with hand counter clickers) that were carrying pollen or not for 5 min. Fanners were defined as bees standing near the entrance, facing the colony interior, with beating wings and slightly raised abdomens. After counting the foragers, the number of fanners were counted using a simple visual scan of the hive entrance area and the inside of the mask. Bearding behavior, scored as occurring or not, was defined as collections of bees clinging to each other, forming a coherent mass outside of the hive.

### Measurement of colonial gas exchange

Colonial gas exchange was measured early in morning, before the heating stress, and at the end of the heat-stress period. Each measurement started with approximately 5 min of measurement of the water, oxygen and carbon dioxide concentrations of the incurrent air to the respiratory mask (Fig. 1; Fig. S1), taken by inserting the subsampling tubes into the outflow from the silica gel column. Once the values had stabilized (usually after 1–2 min), the subsampling tubes were placed into the respiratory mask, and we measured the water, oxygen and carbon dioxide concentrations within the mask for approximately 20 min. After the values had stabilized (usually after 2–3 min), we averaged concentrations of all gases. Then, we remeasured the gas concentrations in the incurrent air to the respiratory mask, as described above. To estimate gas exchange per gram of bee, we divided gas exchange rates by an estimate of the mass of bees in the colony.

To assess gas exchange of a colony of honey bees, we employed an open-flow mask technique (Lighton, 2019). The respiratory mask for the hive was manufactured by the ASU Instrument Design and Fabrication core to fit snugly into entrance of a hive (Fig. 1; Fig. S1). A compressor (Husky 17 liter model 3,320,445) generated pressurized air flow, which was regulated by an Omega mass flow meter at approximately 2 liters min^−1^. From the mass flow meter, air flowed through a column containing silica gel to lower and stabilize air humidity, and then was split into three inlet ports into the hive mask, exiting out the mask opening to the atmosphere. Duct tape was placed over any cracks in the hive (including between hive boxes, top cover and bottom board, and between the respiratory mask and the hive body), ensuring that the hive box was near air-tight and thus that we were able to capture all relevant gas exchange. Air was pulled from the three locations through plastic tubing within the mask into a subsampling system driven by a pump on the Sable Systems FoxBox controlled by the Foxbox mass flow controller at approximately 200 ml min^−1^. Subsampled air was drawn first through a Sable Systems RH-300 water sensor, then a Sable Systems FC-10 oxygen analyzer, followed by a Sable Systems CA-10 carbon dioxide analyzer, and then the FoxBox pump, with air exiting to the atmosphere. Output of each instrument was digitized with a Sable Systems UI2 and recorded to a laptop computer running Sable Systems Expedata, sampling once per second. Using the UI2 and Expedata, we also continuously measured local shaded air temperature and the flow rate of the subsampling system.

#### Gas analyzer calibrations and sensitivity

The RH-300 water sensor (resolution 0.001% relative humidity) was calibrated by injecting small volumes of water into the flow-through system and integrating the peak using Expedata. The difference between incurrent and excurrent (hive) relative humidity always exceeded 0.1%, so the signal-to-noise ratio exceeded 1000. The FC-10 oxygen sensor (resolution 0.001%) was spanned with dry, CO_2_-free air to 20.94% oxygen. The minimum incurrent minus excurrent (hive) oxygen level was 0.008, so the minimum signal-to-noise ratio for oxygen was 8:1. The CA-10 carbon dioxide analyzer (resolution 0.001%) was two-point calibrated with nitrogen gas and a certified CO_2_ standard. The minimum excurrent (hive) minus incurrent carbon dioxide concentration was 0.6%, so a minimal signal-to-noise ratio was 600. The Omega mass flowmeter (resolution ±2%) was calibrated with an SKC soapfilm bubblemeter. Flow rates into the respiratory mask as measured by the Omega mass flow meter varied by approximately 10%, likely because of cycles of the compressor, and these were noted visually from the meter's display and were recorded at a resolution of 0.01 l min^−1^ for each measurement.

#### Equations

We calculated STP gas exchange from our measurements using equations from Lighton (2019):

(1) $$V_{O_{2}}=FR_{i}\;\left(FI_{O_{2}}-\left[\frac{FE_{O_{2}}(1-FI_{O_{2}}-FI_{CO_{2}}-FI_{H_{2}O})}{1-FI_{O_{2}}-FI_{CO_{2}}-FI_{H_{2}O}}\right]\right),$$

(2) $$V_{CO_{2}}=FR_{i}\left(\left[\frac{FE_{CO_{2}}(1-FI_{O_{2}}-FI_{CO_{2}}-FI_{H_{2}O})}{1-FE_{O_{2}}-FE_{CO_{2}}-FE_{H_{2}O}}\right]-FI_{CO_{2}}\right),$$

(3) $$V_{H_{2}O}=FR_{i}\left(\left[\frac{FE_{H_{2}O}(1-FI_{O_{2}}-FI_{CO_{2}}-FI_{H_{2}O})}{1-FE_{O_{2}}-FE_{CO_{2}}-FE_{H_{2}O}}\right]-FI_{H_{2}O}\right),$$

where *V*_O_2__ indicates oxygen consumption by the colony, *V*_CO_2__ is carbon dioxide production and *V*_H_2_O_ is colonial water production. In these equations, measurements of gas exchange (e.g. *V*_O_2__) and flow rate (FR) are in STP ml min^−1^. An i or subscript i indicates incurrent (flowing into the mask) conditions, whereas an e or subscript e indicates conditions in the excurrent air, subsampled from the mask. *F* refers to the fractional content of the gas (e.g. 1%=0.01). The outputs of the oxygen and carbon dioxide analyzers (%) were converted to a fractional content by dividing by 100. The water sensor output was relative humidity. This was converted to kPa by multiplying by the saturation vapor pressure at the temperature of the sensor, and then to a fractional content by dividing by the total barometric pressure. The respiratory exchange ratio was calculated as *V*_CO_2__/*V*_O_2__.

### Measurement of hive contents

For each hive, on the day before the heat stress or early in the morning before heating occurred, we took a digital photograph of each side of each frame using a Canon EOS Rebel T5 camera. If a significant beard formed during the heat-stress measurements, we also took photos immediately after the heat stress before bees could return to the hive so we could accurately assess the number of adults present during the final gas exchange measures. In 2024, we also used frame photographs to measure the area occupied by capped and uncapped honey, pollen and melted wax on each frame for seven colonies.

#### Estimation of the number and mass of bees in a hive

Hives were exposed to smoke and frames were handled gently to minimize bee flight. If the colony exhibited significant bearding, we counted adults immediately after the heat stress; otherwise, the count of bees taken before heat stress was used. In 2022, we counted adult workers with ImageJ, clicking on and marking each individual. In 2024, we used a deep learning method, which we verified against the hand-counted 2022 data for two colonies (results were within 2%).

We estimated the mass of bees in the hive from the adult counts. Adult bees emerge at approximately 0.12 g, gain mass to approximately 0.15 g, and then decline to under 0.09 g after initiation of foraging (Harrison, 1986), so to estimate the mass of all adults we multiplied the number of adults times 0.11 g. We did not count the number of larvae and pupae in these colonies, but for similar colonies in the ASU apiary of the same stock, there were approximately 1.5 larvae per eight adults and 3.5 pupae per eight adults (Chen et al., 2026), so we estimated the number of larvae as 1.5 divided by 8 times the number of adult bees, and the number of pupae as 3.5 divided by 8 times the number of adult bees. Larvae grow from approximately 0.0001 to 0.15 g (Petz et al., 2004), so we estimated the mass of all larvae as number of larvae times 0.075 g. We estimated the mass of all pupae as the number of pupae times 0.135 g. We estimated the mass of bees in each colony by summing the estimated total masses of adult workers, larvae and pupae. This assumes that the mass of eggs is trivial, which seems reasonable given that in similar colonies there was approximately 1 egg per 20 adult workers, and eggs weigh approximately 0.0001 g (Human et al., 2013).

#### Honey and wax contents

We measured honey and wax contents of the colonies in 2024. After photographing frames with the workers, the honey and wax content were measured by gently brushing off the adult bees so that the honey and pollen could be easily observed. The area (cm^2^) occupied by these resources on each frame was measured using the Region of Interest polygon selection tool of ImageJ, tracing each section of each type of cell. The scale was calibrated for each separate image, using the straight-line tool across the top bar of the frame, with a known distance of 45.3 cm, to determine pixel-to-area conversion. Area values were converted to estimated cell counts using a factor of 3.56 cells cm^−2^ (Human et al., 2013). A total of 322 images were analyzed across the seven colonies. Total honey stores were positively correlated with both the estimated bee mass and the number of frames occupied by bees in the colony (Figs S2, S3).

### Quantifying contributions to thermoregulation

#### Rates of physiological and passive heat buffering

We imposed a heat stress on the hives and quantified five potential responses that ameliorate the rise in internal hive temperatures—reduced metabolic heat production, increased evaporative cooling, passive heat storage in bees, and passive heat storage in honey and wax. An Excel sheet with our calculations for each of these mechanisms of heat mitigation is provided (Dataset 2). We estimated the reduction in metabolic heat production by the colony in response to heat stress from the average decrease in oxygen consumption rate (ml s^−1^), converted to watts using an oxygen to joules conversion of 19.56 J ml^−1^ O_2_ (Lighton, 2019), based on the average respiratory exchange ratio of all colonies of 0.69 (see below). We measured the contribution of an increase in evaporative water loss from the increase in water vapor loss induced by the heat stress (ml s^−1^), converting a ml of STP gaseous water to 0.000803 g, and converting to watts assuming a latent heat of water of 2413 J g^−1^ at 37°C, the average brood temperature during the heat trial. We measured rates of passive heat storage in bees, honey and wax from measured temperatures of the hive frames and our estimates of the mass of each of these hive components.

#### Rate of passive heat storage in bees

Stabentheiner et al. (2021) measured bee body and comb temperatures of a hive heated from air temperatures of 14–40°C. Focusing on the higher thermal range overlapping with our study, as environmental air temperatures rose from 30°C to 40°C, body temperatures of bees approximated comb temperatures for both endothermic and ectothermic bees, both on and off the brood comb. Therefore, for this study, we assumed that bee body temperatures can be approximated by the comb temperatures that we measured. Passive heat storage in bees (*B*, in J) was calculated assuming that the initial and final temperatures of the larvae and pupae were the average of the brood center and brood edge temperatures; their temperatures rose by an average of 3.5°C during the heat stress. Adult bees tend to congregate over the brood but also distribute throughout the hive on frames with pollen or honey. Therefore, for adult bees, we assumed that 25% were in the brood center, 25% in the brood edge area and 50% in the hive periphery; with this calculation, the average body temperature of an adult bee in the hive increased by 7.5°C. Passive heat storage (J) in the bodies of each life stage of bees was then calculated as:

(4) $$B=\Delta T_{B}\times M\times SH_{B},$$

where Δ*T* is the estimated rise in temperature of that life stage during the heat stress (^o^C) and *M* is the total mass of that life stage (g). To our knowledge, the specific heat of honey bees (SH_B_) has not yet been measured, so we estimated this using the equation of Giering et al. (1996):

(5) $$SH_{B}=4.19\times (0.37+0.63W),$$

where SH_B_ is in J g^−1^ °C^−1^ and *W* is water content as a decimal fraction. Water content of larvae and pupae are reported as 0.76–0.84, and for adults from 0.69 to 0.82 (Hrassnigg and Crailsheim, 2005). We used the mean water content from these ranges (0.78), and estimated the specific heat of honey bees as 3.61 J °C^−1^ g^−1^. We summed the estimated heat stores for each life stage to calculate total heat storage in the bodies of bees during the heat stress, and then divided by the average heating time in seconds to calculate the rate of passive heat storage in watts. Confidence intervals for heat storage in bees were calculated from the variation in total masses of bees, as this was the major factor causing variation among colonies.

#### Rate of passive heat storage in honey

As honey is located outwards from the brood edge into the periphery, the rate of passive heat storage in honey (*H*, in W) was calculated by assuming that average honey temperature is the mean of brood edge and hive periphery temperatures:

(6) $$H=(\Delta T_{p}+\Delta T_{\mathrm{be}})/2)\times M_{h}\times SH_{h}\times T_{h}^{-1},$$

where Δ*T*_p_ is the rise in temperature of the hive periphery during the heat stress, Δ*T*_be_ is the rise in temperature of the brood edge during the heat stress, *M*_h_ is the mass of honey in grams, and *T*_h_ is the heating time (the average duration of the heat stress exposure in seconds). The mass of honey in a colony was calculated by summing the measured cm^2^ of total honey (capped and uncapped), dividing by 2323 cm^2^ (both sides of a frame) and multiplying by 2370 g of honey per frame (average mass of honey in a full honey frame in our bee yard, calculated from the difference in mass of full and extracted honey frames). We used a specific heat for honey of 2.049 J g^−1^ °C^−1^, which is the average value reported by Sopade et al. (2006) at 35°C and 45°C.

#### Rate of passive heat storage in wax

We calculated the average number of frames per colony from the number of boxes×10 frames per box×0.9 as cells had been constructed on most frames in these hives. We used a specific heat for beeswax of 2.08 J g^−1^ °C^−1^ (Abdulmunem et al., 2024). We modeled wax temperature change assuming that wax temperatures are identical to brood temperatures in the brood area, and that wax temperatures are identical to honey temperatures in the remainder of the hive. We calculated the mass of wax in the brood area from the average number of larvae+pupae in our colonies, the number of frames occupied by this amount of brood, and the mean mass of wax per frame (284 g, measured for other colonies in our apiary).

### Statistics

Effects of the heating stress on hive temperatures, behavior and gas exchange were tested with Wilcoxon signed rank tests as these were all paired data and not all of these data satisfied assumptions of normality. For effects of heating stress on bearding behavior (nominal data), we used a chi-squared test with Yates correction for small sample sizes. All statistics were performed with GraphPad Prism v10 for MacIntosh, with a *P*-value for significance set at 0.05.

## RESULTS

### Natural diurnal variation in hive temperatures

**Fig. 2.**
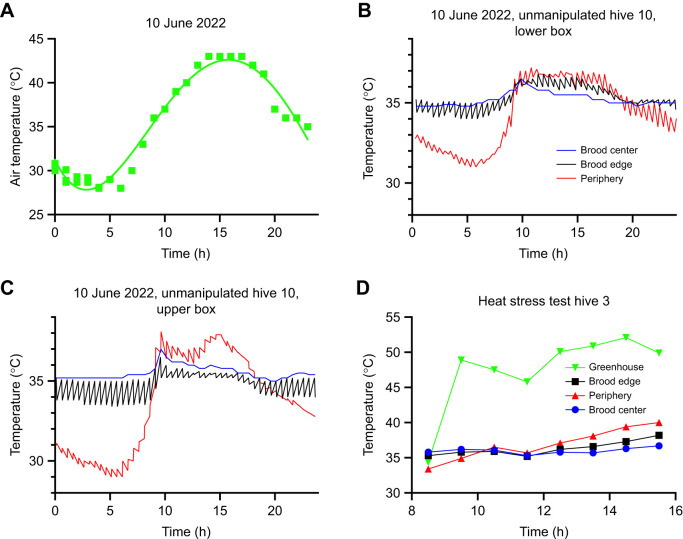
**Comparison of natural diurnal variation in hive temperatures with those occurring during an experimental heat stress test.** (A–C) Diurnal temperature variation on a summer day in an unmanipulated honey bee colony of the same average size and heat storage capacity as colonies used in the heat stress test. (A) Shaded air temperature (from Mesa Gateway airport, approximately 1 km from the bee yard) versus time of day (military time). Fit-line is air temperature=31.25–2.624*T*+0.585*T*^2^–0.0309*T*^3^+0.000464*T*^4^ (*r*^2^=0.97). (B) Brood center, brood edge and hive periphery for the lower hive box. (C) Same for the upper hive box. (D) Time course of air, brood and periphery temperatures during heat stress. Both the unmanipulated hive 10 and the heat-stressed hive 3 had approximately 14,000 worker bees, similar to the average population size in our heat stress tests.

Bees allow internal hive temperatures to cool at night, especially in the hive periphery (Chen et al., 2026), and such nocturnal cooling can prepare the hive to passively absorb diurnal heat with less physiological stress. Measurements of internal temperatures of a hive of similar adult population and pollen content as the average hive studied here show that temperatures in the hive periphery, where most honey is located, cycle by more than 5°C in the lower box and by more than 9°C in the upper box (Fig. 2A–C). Most of this diurnal cycling occurs because bees closely thermoregulate the brood but not the periphery at night (Fig. 2B,C).

### Effect of heat stress on nest temperatures, bee behavior and colonial gas exchange

**Fig. 3.**
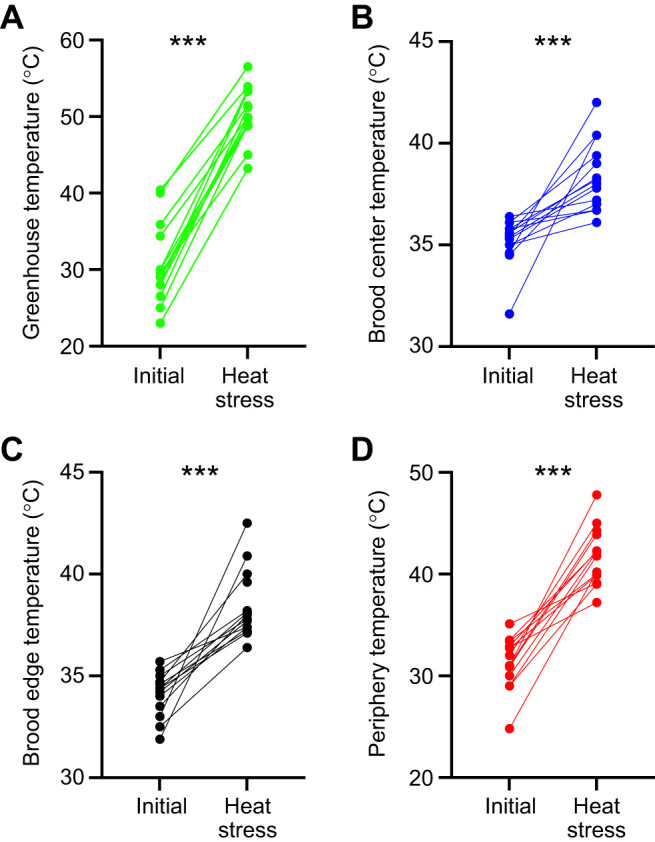
**Effect of heat stress on temperatures outside and inside the colony.** (A) Greenhouse temperature was elevated by 20.2±3.70°C from starting values of 29.1±1.10 to 49.8±1.40°C. (B) Brood center temperatures rose by 2.5±1.4°C from 35.4±0.4 to 38.0±1.0°C. (C) Brood edge temperatures rose by 3.5±0.80°C from 34.4±0.60 to 37.8±0.60°C. (D) Hive periphery temperatures rose by 10.0±3.00°C from 32.0±1.40 to 42.2±2.10°C. Asterisks indicate that all comparisons are significant by Wilcoxon signed rank tests (***P<0.001; *n*=15 colonies for each test). In this and subsequent figures, the median is reported, followed by the median absolute deviation in parentheses.

Raising the greenhouse temperature to approximately 50°C produced slow but steady increases in the temperatures of the brood center, edge and hive periphery (Fig. 2D; Figs S4–S6). Exposure to an air temperature of 50°C caused brood temperatures to rise from approximately 35°C to 38°C within 2–7 h (Fig. 3). Simultaneously, temperatures in the hive periphery rose by more than 10°C (Fig. 3).

**Fig. 4.**
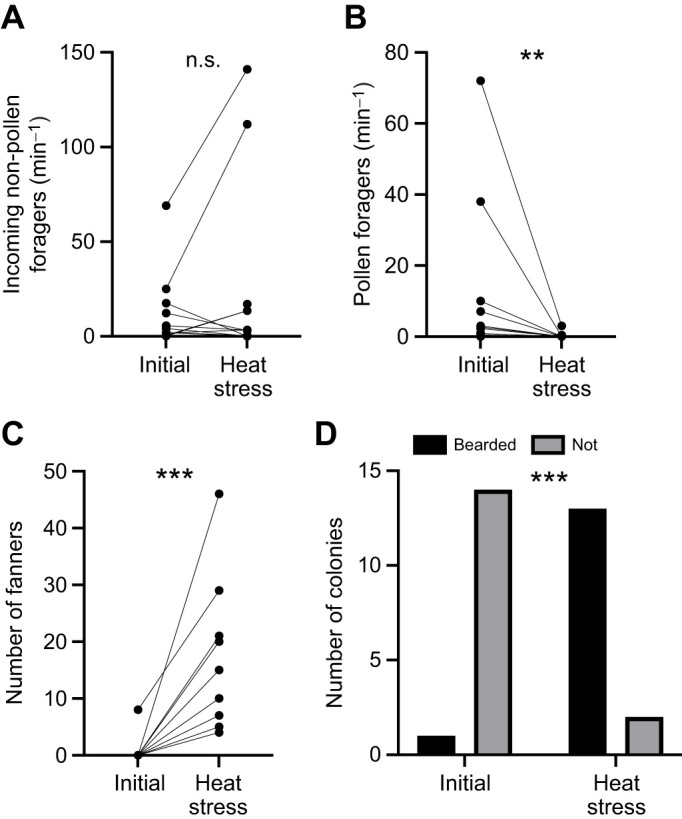
**Effects of heat stress on colony behavior.** (A) There was no significant effect of the heat stress on the rate of incoming nonpollen foragers (initial=4.2±3.00, final=3.2±3.05; change=−1.8±5.4; Wilcoxon signed rank test, *P*=0.863, *n*=12 colonies). (B) The rate of incoming pollen foragers fell by 2.7±2.45 from 2.7±2.40 to 0±0; Wilcoxon signed rank test, *P*=0.002, *n*=12 colonies. (C) Visible fanners increased by 10±5 from 0±0 to 10±5; Wilcoxon signed rank test, ***P<0.001, *n*=15 colonies. (D) Heat stress induced bearding behavior (chi-squared test with Yates correction χ^2^_1_=16.2, ***P<0.001, *n*=15 colonies).

The heat stress strongly suppressed pollen foraging, but had no effect on the number of incoming non-pollen foragers (Fig. 4). Fanning behavior was strongly increased, and most colonies exhibited bearding (Fig. 4).

**Fig. 5.**
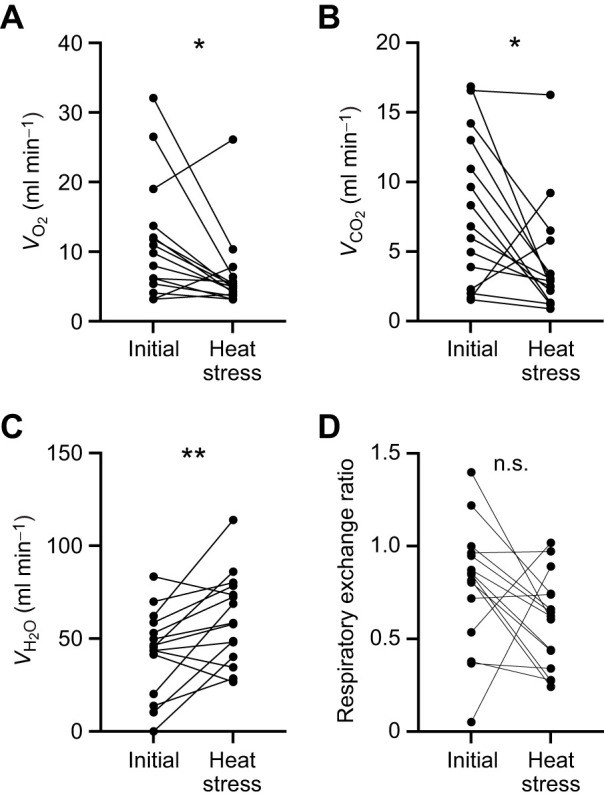
**Effects of heat stress on gas exchange of honey bee colonies.** (A) Oxygen consumption rates decreased by a median of 3.0±3.37 from medians of 9.8±3.92 to 5.0±2.71 ml min^−1^ (Wilcoxon signed rank test, *P=0.021, *n*=15). (B) Median carbon dioxide production decreased by 2.9±4.22 from 6.8±4.52 to 2.9±1.58 ml min^−1^ (Wilcoxon signed rank test, *P=0.015). (C) Median water loss increased by 14.8±23.46 from 45.4±13.25 to 58.4±18.18 ml min^−1^ (Wilcoxon signed rank test, **P=0.007). (D) Median respiratory exchange ratio did not change significantly (n.s., Wilcoxon signed rank test, *P*=0.083), though there was a trend toward a lower value after heat stress (initial=0.85±0.132, final=0.62±0.183, change=−0.31±0.269). The average respiratory exchange ratio pooling initial and final values was 0.69. *N*=15 for each variable and test.

During heat stress, oxygen consumption and carbon dioxide production rates were approximately 50% lower and evaporative water loss approximately 29% higher than under initial conditions (Fig. 5A–C). The respiratory exchange ratio was statistically unchanged (Fig. 5D).

### Nest thermoregulation patterns and mechanisms

**Fig. 6.**
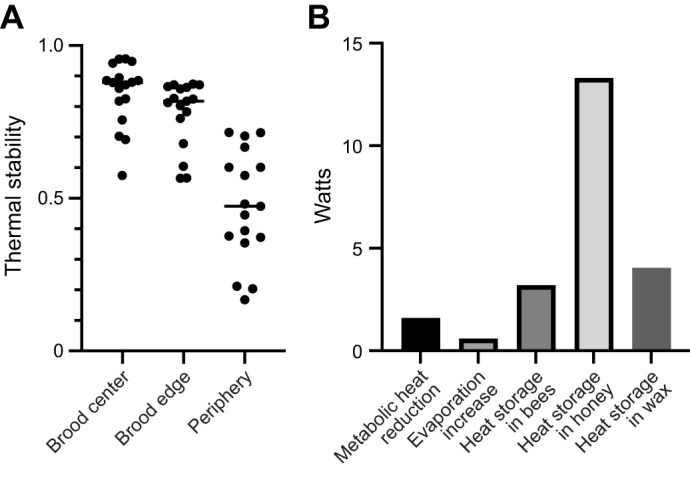
**Colonial thermoregulation and its mechanisms in an experimentally heat-stressed honey bee colony.** (A) Thermal stability, defined as 1–(change in region temperature/change in enclosure temperature). Median thermal stability of the brood center was 0.88±0.062, for the brood edge was 0.82±0.046 and for hive periphery was 0.47±0.424. *N*=15 for each variable. (B) Rates of mechanisms of heat buffering during the heat stress were metabolic heat reduction (1.6 W), increased evaporative heat loss (0.6 W), heat storage in bees (3.2 W), heat storage in honey (13.3 W) and heat storage in wax (4.0 W). *N*=15 for each variable.

We assessed colonial thermoregulatory precision on a scale from 0 (no regulation) to 1 (perfect thermoregulation) calculated as 1–(change in hive temperature/change in enclosure temperature). Colonies regulated brood center and brood edge temperatures well, but not perfectly, thermoregulating with a precision of 0.88 and 0.82, respectively. Hive periphery temperatures were much less accurately thermoregulated, with a precision of 0.47 (Fig. 6A).

Passive heat storage was by far the dominant mechanism contributing to thermal stability of the colony during exposure to heat stress (Fig. 6B), with heat storage in honey estimated to be 13.3±4.74 W, heat storage in wax to be 4±0.68 W, and heat stored in the bodies of bees to be 3.2±0.71 W. Surprisingly, the least quantitatively important mechanism of thermal stability was the increase in evaporative cooling, accounting for 0.6±0.31 W of enhanced heat loss in response to the heat stress, whereas metabolic heat production decreased by 1.6±1.33 W.

## DISCUSSION

Passive heat storage was the predominant mechanism contributing to thermal stability of our honey bee colonies when exposed to transient heating, as often occurs diurnally during heat waves. Passive heat storage mechanisms will vary with colony size, as worker number, the number of combs, and the area of wax and honey tend to be positively correlated (Figs S2, S3), providing selective advantage for larger colonies. Incipient colonies with minimal wax and honey, or strains that store less honey, such as Africanized bees (Winston, 1992), will have less passive heat storage, be more dependent on the behavioral and physiological mechanisms of thermoregulation, and may experience greater thermal variation and negative fitness effects of heat waves (Chen et al., 2026). Wild honey bees select relatively large cavity volumes for nests, and place brood in the center of the honey and pollen stores (Seeley and Morse, 1976), improving insulation and the capacity for passive heat storage to aid in thermal stability. The importance of passive nest design for thermal stability of social insect nests has long been appreciated (Jones and Oldroyd, 2007), and our study allows quantification of the importance of passive heat stores in ameliorating effects of diurnal heat stress.

It is important to note that our study was not designed to create a formal heat budget for a honey bee colony. Assessment of radiative, conductive and convective exchanges, including rates of air flow and air temperatures through the entrance, and how these are affected by environmental conditions and hive construction are needed to do so. Even for these specific conditions, our heat budget is based on some assumptions that require further research. One important point is that we assumed a constant distribution of bees on the comb, with bee temperatures predictable from our thermal sensors. This may underestimate the rise in adult bee temperature, as bees are reported to disperse away from the brood area during heat stress and move toward the periphery (Stabentheiner et al., 2021). If 50% of the bees dispersed from the brood area into the periphery, this could approximately double the quantity of heat stored in the bodies of bees, exceeding the heat storage in wax, but not the storage in honey (Table S1).

Because the passive heat-storage estimates are linearly proportional to mass and temperature change, uncertainty in these parameters scales the estimated contribution of each storage component proportionally (Table S1). Thus, although even relatively large changes in bee or honey mass will alter the absolute wattage estimates, these are unlikely to change the qualitative conclusion that passive heat storage, especially in honey and wax, dominates whole-colony heat buffering during a mid-day diurnal heat challenge. For example, doubling the estimated heat storage in bees under an alternative bee-distribution scenario would make bee bodies a larger passive heat store than wax, but honey would remain the largest single heat-storage component, and passive heat storage would still exceed the combined contributions of metabolic suppression and increased evaporative cooling.

More detailed assessment of the distribution of wax and honey temperatures in a hive would also improve the accuracy of our heat budget. Direct measurement of bee and comb temperatures would easily be possible in an observation hive (Stabentheiner et al., 2021), but these would likely differ from conditions in an intact hive, so the most obvious improvement would be to rerun experiments such as ours with substantially more and well-distributed thermal sensors. Better temporal resolution of changes in metabolic rates, water loss and internal temperatures could also improve our understanding of the heat buffering mechanisms in honey bee colonies.

The discovery that increases in evaporative cooling account for only approximately 3% of the whole-colony thermal stability in response to a heat-stress event is surprising given prior paradigms of honey bee colonial thermoregulation, and has important implications for understanding heat mitigation in bees and other social insects. First, although these data show that evaporative heat loss is not a major component of whole-colony heat buffering, it must be very important for brood temperature regulation. As our data show, the brood area is much more tightly thermoregulated than the outer regions of the hive, and this must be due to behaviorally localized mechanisms including evaporative cooling. However, our findings suggest that the reduction of metabolic heat production is at least as important for whole-colony thermal regulation as the increase in evaporative cooling. During heat stress, bees leave the brood area and those remaining reduce endothermic behavior as indexed by thoracic temperatures, with the decrease in metabolic rates predicted from bee body temperatures similar to our direct measures (Stabentheiner et al., 2021).

Honey bee colonies allow internal hive temperatures to cool at night, especially in the hive periphery (Fig. 2; Chen et al., 2026), and this behavior helps protect the colony from diurnal heat stress just as high thermal mass can passively stabilize circadian cycles in building temperatures. Honey that is cool in the morning can serve as a passive buffer for heat storage through the day, greatly reducing the need for evaporative cooling and contributing to minimization of heat stress exposure. In contrast, the high thermal inertia of the hive means that the colony will continue to experience heat stress even after sundown from peripheral honey warmed to over 38°C; given that bees cannot collect water at night, this could pose a significant threat to brood temperatures. This may partially explain why bearding behavior for honey bee colonies is most extensive at night, even when air temperatures are cooler (Rowe et al., 2026). The 7°C average diurnal cycle in honey temperatures (Fig. 2) during the Arizona desert summer is similar to what we observed with our experimental heat stress, suggesting that passive heat release and absorption is the primary mechanism contributing to thermal stability in honey bee colonies exposed to naturally occurring circadian variation in desert temperatures.

Many important questions remain concerning the role of behavior and different types of dangers associated with overheating. Bearding behavior can contribute to thermoregulation by lowering the metabolic heat production within the hive by adult bees, and 87% of the colonies exposed to the heating challenge exhibited some bearding behavior (Fig. 3). However, excessive bearding behavior may imperil the colony by hindering the evaporative cooling needed for the nest, and possibly by reducing ventilation needed to prevent regional hot spots. Anecdotally, in one colony (not included in the dataset) for which we failed to stop heating when the brood temperature reached 38°C, over 90% of adult bees left the hive, while the queen remained inside within the 42°C brood area, resulting in her death within a week. Several other colonies experienced wax melting and a ‘rain’ of hot honey from uncapped honey cells, coating and killing workers, illustrating the perils of abandoning the task of cooling the periphery. Although older wax can be resistant to melting at temperatures over 60°C, our results support experimental findings that newly produced wax can fail at temperatures near 50°C (Abdulmunem et al., 2024).

Depression of metabolic rates in the heat is an important mechanism of thermoregulation in colonial honey bees, as it is in other endotherms. Prior measurements of resting metabolic rates of honey bees appear to overestimate heat generation inside a full-sized, functioning hive, likely due to stress effects. ‘Resting’ individual bees in metabolic chambers at air and body temperatures of 35°C have been reported to have metabolic rates of 80 mW g^−1^ (Harrison and Fewell, 2002). Kronenberg and Heller's measurements of frames of bees reported metabolic rates of 80 and 20 mW g^−1^ at air temperatures of 20°C and 35°C, respectively (Kronenberg and Heller, 1982). We calculated metabolic rates of 2.6 and 1.3 mW g^−1^ at enclosure air temperatures of 30°C and 50°C, respectively. Inclusion of brood in the measurements likely reduces the calculated mass-specific metabolic rate. We estimated brood to account for 42% of the bee mass in an average colony, and larvae and pupae generally have significantly lower metabolic rates than adult social insects (Waters and Harrison, 2012). Our estimates of bee metabolic rates in hives are 2–3 times the metabolic rate of a generic resting 0.1 g insect at a body temperature of 35°C (0.05 mW g^−1^; Chown et al., 2007), which seems reasonable given that bees in hives are performing a range of activities.

Our findings demonstrate that honey bee colonies are vulnerable to air temperatures of 50°C, which happen occasionally but are predicted to occur more frequently owing to climate change (Donat et al., 2020; Kornhuber et al., 2024). However, extrapolation to other environments and conditions should be made cautiously. First, our manipulation of putting hives in a greenhouse likely reduced convective heat loss from the hive, exacerbating the heat stress. However, our hives were sheltered from solar radiation, which can greatly increase heat loading. Heat stress effects depend on their duration and frequency, as well as windspeed and radiation from the sun and environment (Harvey et al., 2023; Johnson et al., 2023; Kingsolver et al., 2021). Thus, the relationship between air temperature and honey bee colonial fitness is complex. Most honey bee brood can survive brief periods of 38°C or higher (Medina et al., 2018). However, heat waves with maximal air temperatures over 40°C cause brood temperatures to exceed those for optimal development for multiple hours each day, resulting in smaller worker populations (Chen et al., 2026). These findings suggest that negative fitness effects of heat waves on bees can occur at air temperatures well below 50°C. As of yet, we lack the needed studies of the effects of various durations and frequencies of heat stress on honey bee brood development, adult lifespans, and drone and queen reproductive traits to accurately predict the full scope of fitness effects from heat waves.

Our finding of failed brood thermoregulation at 50°C is arguably contradicted by Wohlgemuth (1957), who reported that, after several hours, hives surrounded with 50°C air were able to regulate brood temperatures at equilibrium temperatures of 36.5°C. However, the air outside the open entrance for Wohlgemuth's colonies was approximately 10°C, likely aiding in the colony's capacity to thermoregulate. This scenario, however, is unlikely to occur in nature. In our study, air entering the hive through the entrance was also cooler than 50°C (generally it was 35–40°C), likely reducing the experienced heat stress.

Might colonies have been better able to achieve thermal stability if provided more time? We measured the internal temperatures for more than 10 h for three colonies exposed to 50°C. Two colonies were able to stabilize brood center temperatures, and the third showed steady increases in brood center temperature over time (Figs. S4). Temperatures at the brood edge increased steadily for all three colonies (Fig. S5) and periphery temperatures rose steadily for two of the colonies (Fig. S6), suggesting that the heat load was progressively overwhelming the thermoregulatory capacities of these colonies (Figs S4-S6).

Given the demonstrated vulnerabilities of honey bee colonies to heat, it will be important for beekeepers in affected regions to develop mitigation strategies. One clear conclusion is that strategies that reduce heat loading and add capacities for heat storage will make colonies less vulnerable to lethal overheating. These include managing colonies to have larger populations and store more honey, as larger colonies have significantly greater thermoregulatory capacities (Chen et al., 2026). Providing shade and using construction materials that provide thermal mass also could be helpful. However, it is also possible that commercial beekeepers in regions experiencing such high temperatures may need to relocate their apiaries or temporarily move hives into cooled buildings.

Many insects beyond honey bees are important pollinators and it will be critical to assess the vulnerability of insect pollinator nests to heat stress more broadly. Ground-nesting species would seem to be less vulnerable owing to the essentially infinite heat capacity of the ground. However, the nests of many insect pollinators (bumblebees, many solitary bees) are relatively shallow, and may be vulnerable to heating (Hines, 2026; Kevan et al., 2024; Michener, 2007). Other above-ground nesters (e.g. other species of *Apis*, leafcutter bees, some wasps) may be particularly vulnerable to heat stress in their exposed, poorly insulated nests. Many individual insects have upper critical maximal temperatures near or below 50°C (Johnson et al., 2023). The vagility of adult forms allows them to find microhabitats to avoid the hottest conditions and survive. However, nests are less mobile and therefore more vulnerable. Further research on the upper thermal limits of insect nests will be critical to predict when warming-induced heat waves may begin to have strong negative effects on pollinator populations. Quantifying the limits and mechanisms of nest thermoregulation reveals that even highly social insects possess finite heat buffering capacities, constraints that will likely increasingly shape pollinator persistence.

## Supplementary Material

10.1242/jexbio.252357_sup1 Supplementary information

Dataset 1. All data used for creation of the graphs and statistical analyses in this manuscript are available in this separate Excel file.

Dataset 2. This file shows the calculations used for estimating the contributions to thermoregulation during the heat stress.

Dataset 3. This file shows the calculation of median absolute differences for all of the variables.

## References

[JEB252357C1] Abdulmunem, A. R., Mazali, I. I., Samin, P. M. and Sopian, K. (2024). Beeswax as a sustainable thermal energy storage material: experimental thermal assessment in solar air heater. *J. Energy Storage* 103, 114398. 10.1016/j.est.2024.114398

[JEB252357C2] Bonoan, R. E., Goldman, R. R., Wong, P. Y. and Starks, P. T. (2014). Vasculature of the hive: heat dissipation in the honey bee (*Apis mellifera*) hive. *Naturwissenschaften* 101, 459-465. 10.1007/s00114-014-1174-2 24760416

[JEB252357C3] Bordier, C., Dechatre, H., Suchail, S., Peruzzi, M., Soubeyrand, S., Pioz, M., Pélissier, M., Crauser, D., Conte, Y. L. and Alaux, C. (2017). Colony adaptive response to simulated heat waves and consequences at the individual level in honeybees (*Apis mellifera*). *Sci. Rep.* 7, 3760. 10.1038/s41598-017-03944-x 28630407 PMC5476575

[JEB252357C4] Chadwick, L. E. (1931). Ventilation of the hive. *Glean. Bee Cult.* 59, 356-358.

[JEB252357C5] Chen, J., Fisher, A., DeGrandi-Hoffman, G., Ozturk, C., Smith, B. H., Fewell, J. H., Kang, Y., Maxwell, K., Overcash, K., Chahal, K. et al. (2026). Negative effects of excessive heat on colony thermoregulation and population dynamics in honey bees. *Ecol. Evol. Physiol.* 99, 1-18. 10.1086/739493 41774888

[JEB252357C6] Chown, S. L., Marais, E., Terblanche, J. S., Klok, C. J., Lighton, J. R. B. and Blackburn, T. M. (2007). Scaling of insect metabolic rate is inconsistent with the nutrient supply network model. *Funct. Ecol.* 21, 282-290. 10.1111/j.1365-2435.2007.01245.x

[JEB252357C7] Donat, M. G., Sillmann, J. and Fischer, E. M. (2020). Changes in climate extremes in observations and climate model simulations. From the past to the future. In *Climate Extremes and Their Implications for Impact and Risk Assessment* (ed. J. Sillmann, S. Sippel and S. Russo), pp. 31-57. Elsevier.

[JEB252357C8] Gallai, N., Salles, J.-M., Settele, J. and Vaissière, B. E. (2009). Economic valuation of the vulnerability of world agriculture confronted with pollinator decline. *Ecol. Econ.* 68, 810-821. 10.1016/j.ecolecon.2008.06.014

[JEB252357C9] Giering, K., Lamprecht, I. and Minet, O. (1996). Specific heat capacities of human and animal tissues. *Proc. SPIE* 2624, 188-197. 10.1117/12.229547

[JEB252357C10] Goulson, D. (2022). *Silent Earth: Averting the Insect Apocolypse*. New York: Harper Collins Publishers.

[JEB252357C11] Groh, C., Tautz, J. and Rössler, W. (2004). Synaptic organization in the adult honey bee brain is influenced by brood-temperature control during pupal development. *Proc. Natl Acad. Sci. USA* 101, 4268-4273. 10.1073/pnas.0400773101 15024125 PMC384730

[JEB252357C12] Hanley, N., Breeze, T. D., Ellis, C. and Goulson, D. (2015). Measuring the economic value of pollination services: principles, evidence and knowledge gaps. *Ecosyst. Serv.* 14, 124-132. 10.1016/j.ecoser.2014.09.013

[JEB252357C13] Harrison, J. M. (1986). Caste-specific changes in honeybee flight capacity. *Physiol. Zool.* 59, 175-187. 10.1086/physzool.59.2.30156031

[JEB252357C14] Harrison, J. F. and Fewell, J. H. (2002). Environmental and genetic influences on flight metabolic rate in the honey bee, *Apis mellifera*. *Comp. Biochem. Physiol. A* 133, 323-333. 10.1016/S1095-6433(02)00163-0 12208303

[JEB252357C15] Harvey, J. A., Heinen, R., Gols, R. and Thakur, M. P. (2020). Climate change-mediated temperature extremes and insects: from outbreaks to breakdowns. *Glob. Change Biol.* 26, 6685-6701. 10.1111/gcb.15377 PMC7756417 33006246

[JEB252357C16] Harvey, J. A., Tougeron, K., Gols, R., Heinen, R., Abarca, M., Abram, P. K., Basset, Y., Berg, M., Boggs, C., Brodeur, J. et al. (2023). Scientists’ warning on climate change and insects. *Ecol. Monogr.* 93, e1553. 10.1002/ecm.1553

[JEB252357C17] Hazelhoff, E. H. (1954). Ventilation in a bee-hive during summer. *Physiol. Comp.* 3, 343-364.

[JEB252357C18] Himmer, A. (1927). Ein Beitrag zur Kenntnis des Warmehaushalts im Nestrau Socialer Hauteflugler. *Z. Vgl. Physiol.* 5, 375-389. 10.1007/BF00338020

[JEB252357C19] Hines, H. M. (2026). Comparative evolution of social and ecological traits in bumble bees. *Annu. Rev. Entomol.* 71, 189-207. 10.1146/annurev-ento-121423-013636 41021634

[JEB252357C20] Hrassnigg, N. and Crailsheim, K. (2005). Differences in drone and worker physiology in honeybees (*Apis mellifera)*. *Apidologie* 36, 255-277. 10.1051/apido:2005015

[JEB252357C21] Human, H., Brodschneider, R., Dietemann, V., Dively, G., Ellis, J. D., Forsgren, E., Fries, I., Hatjina, F., Hu, F.-L., Jaffé, R. et al. (2013). Miscellaneous standard methods for *Apis mellifera* research. *J. Apic. Res.* 52, 1-53. 10.3896/IBRA.1.52.4.10

[JEB252357C22] Jhawar, J., Davidson, J. D., Weidenmüller, A., Wild, B., Dormagen, D. M., Landgraf, T., Couzin, I. D. and Smith, M. L. (2023). How honeybees respond to heat stress from the individual to colony level. *J. R. Soc. Interface* 20, 20230290. 10.1098/rsif.2023.0290 37848056 PMC10581772

[JEB252357C23] Johnson, M. G., Glass, J. R., Dillon, M. E. and Harrison, J. F. (2023). How will climatic warming affect insect pollinators? In *Advances in Insect Physiology*, Vol. 64 (ed. J. F. Harrison), pp. 1-115. Academic Press.

[JEB252357C24] Jones, J. C. and Oldroyd, B. P. (2007). Nest thermoregulation in social insects. In *Advances in Insect Physiology*, vol. 33 (ed. S. J. Simpson), pp. 153-191. London: Academic Press.

[JEB252357C25] Kevan, P. G., Rasmont, P. and Martinet, B. (2024). Thermodynamics, thermal performance and climate change: temperature regimes for bumblebee (*Bombus* spp.) colonies as examples of superorganisms. *Front. Bee Sci.* 2, 1351616. 10.3389/frbee.2024.1351616

[JEB252357C26] Kingsolver, J. G., Moore, M. E., Augustine, K. E. and Hill, C. A. (2021). Responses of *Manduca sexta* larvae to heat waves. *J. Exp. Biol.* 224, jeb236505. 10.1242/jeb.236505 34424973

[JEB252357C27] Kornhuber, K., Bartusek, S., Seager, R., Schellnhuber, H. J. and Ting, M. (2024). Global emergence of regional heatwave hotspots outpaces climate model simulations. *Proc. Natl Acad. Sci. USA* 121, e2411258121. 10.1073/pnas.2411258121 39589877 PMC11626130

[JEB252357C28] Kronenberg, F. and Heller, H. C. (1982). Colonial thermoregulation in honey bees (*Apis mellifera)*. *J. Comp. Physiol. B* 148, 65-76. 10.1007/BF00688889

[JEB252357C29] Kühnholz, S. and Seeley, T. D. (1997). The control of water collection in honey bee colonies. *Behav. Ecol. Sociobiol.* 41, 407-422. 10.1007/s002650050402

[JEB252357C30] Lighton, J. R. B. (2019). *Measuring Metabolic Rates: A Manual for Scientists*. New York: Oxford University Press.

[JEB252357C31] Lindauer, M. (1954). Temperaturegulierung und Wasserhaushalt im Bienenstaat. *Z. Vgl. Physiol.* 36, 391-432. 10.1007/BF00345028

[JEB252357C32] Lindauer, M. (1955). The water economy and temperature regulation of the honeybee colony. *Bee World* 36, 81-92. 10.1080/0005772X.1955.11094876

[JEB252357C33] Lu, Y., Hong, W., Wu, W., Zhang, J., Li, S., Xu, B., Wei, K. and Liu, S. (2025). The impact of temperature regulation measures on the thermodynamic characteristics of bee colonies based on finite element simulation. *Biosyst. Eng.* 250, 306-316. 10.1016/j.biosystemseng.2025.01.012

[JEB252357C34] Marx, W., Haunschild, R. and Bornmann, L. (2021). Heat waves: a hot topic in climate change research. *Theor. Appl. Clim.* 146, 781-800. 10.1007/s00704-021-03758-y PMC8414451 34493886

[JEB252357C35] McAfee, A., Chapman, A., Higo, H., Underwood, R., Milone, J., Foster, L. J., Guarna, M. M., Tarpy, D. R. and Pettis, J. S. (2020). Vulnerability of honey bee queens to heat-induced loss of fertility. *Nat. Sustain.* 3, 367-376. 10.1038/s41893-020-0493-x

[JEB252357C36] Medina, R. G., Paxton, R. J., De Luna, E., Fleites-Ayil, F. A., Medina, L. A. M. and Quezada-Euán, J. J. G. (2018). Developmental stability, age at onset of foraging and longevity of Africanized honey bees (*Apis mellifera* L.) under heat stress (Hymenoptera: Apidae). *J. Therm. Biol.* 74, 214-225. 10.1016/j.jtherbio.2018.04.003 29801630

[JEB252357C37] Meehl, G. A. and Tebaldi, C. (2004). More intense, more frequent, and longer lasting heat waves in the 21st century. *Science* 305, 994-997. 10.1126/science.1098704 15310900

[JEB252357C38] Michener, C. D. (2007). *The Bees of the World*. Baltimore, MD: Johns Hopkins University Press.

[JEB252357C39] Mitchell, D. (2016). Ratios of colony mass to thermal conductance of tree and man-made nest enclosures of *Apis mellifera*: implications for survival, clustering, humidity regulation and *Varroa destructor*. *Int. J. Biometeorol.* 60, 629-638. 10.1007/s00484-015-1057-z 26335295

[JEB252357C40] Ostwald, M. M., Smith, M. L. and Seeley, T. D. (2016). The behavioral regulation of thirst, water collection and water storage in honey bee colonies. *J. Exp. Biol.* 219, 2156-2165. 10.1242/jeb.139824 27445400

[JEB252357C41] Parmesan, C., Root, T. L. and Willig, M. R. (2000). Impacts of extreme weather and climate on terrestrial biota. *Bull. Am. Meteorol. Soc.* 81, 443-450. 10.1175/1520-0477(2000)081<0443:IOEWAC>2.3.CO;2

[JEB252357C42] Perkins-Kirkpatrick, S., Barriopedro, D., Jha, R., Wang, L., Mondal, A., Libonati, R. and Kornhuber, K. (2024). Extreme terrestrial heat in 2023. *Nat. Rev. Earth Environ.* 5, 244-246. 10.1038/s43017-024-00536-y

[JEB252357C43] Petz, M., Stabentheiner, A. and Crailsheim, K. (2004). Respiration of individual honeybee larvae in relation to age and ambient temperature. *J. Comp. Physiol. B* 174, 511-518. 10.1007/s00360-004-0439-z 15278398

[JEB252357C44] Rowe, E. B., Prathibha, P., Marting, P. R. and Smith, M. L. (2026). Honey bee beards: internal and external factors driving mass thermoregulatory evacuation. *Insectes Soc.* 73, 201-211. 10.1007/s00040-025-01046-w

[JEB252357C45] Seeley, T. D. and Morse, R. A. (1976). The nest of the honey bee (*Apis mellifera* L.). *Insectes Soc.* 23, 495-512. 10.1007/BF02223477

[JEB252357C46] Simpson, J. (1961). Nest climate regulation in honey bee colonies. *Science* 133, 1327-1333. 10.1126/science.133.3461.1327 17744947

[JEB252357C47] Sopade, P. A., Halley, P. J. and D'arcy, B. R. (2006). Specific heat capacity of Australian honeys from 35 to 165°C as a function of composition using differential scanning calorimetry. *J. Food Process. Preserv.* 30, 99-109. 10.1111/j.1745-4549.2006.00051.x

[JEB252357C48] Southwick, E. E. (1985). Allometric relations, metabolism and heat conductance in clusters of honeybees at cool temperatures. *J. Comp. Physiol.* 156, 143-149. 10.1007/BF00692937

[JEB252357C49] Southwick, E. E. and Moritz, R. F. A. (1987). Social control of air ventilation in colonies of honey bees, *Apis mellifera*. *J. Insect Physiol.* 33, 623-626. 10.1016/0022-1910(87)90130-2

[JEB252357C50] Stabentheiner, A., Kovac, H., Mandl, M. and Käfer, H. (2021). Coping with the cold and fighting the heat: thermal homeostasis of a superorganism, the honeybee colony. *J. Comp. Physiol. A* 207, 337-351. 10.1007/s00359-021-01464-8 PMC8079341 33598719

[JEB252357C51] Starks, P. T. and Gilley, D. C. (1999). Heat shielding: a novel method of colonial thermoregulation in honey bees. *Naturwissenschaften* 86, 438-440. 10.1007/s001140050648 10501692

[JEB252357C52] Wagner, D. L., Grames, E. M., Forister, M. L., Berenbaum, M. R. and Stopak, D. (2021). Insect decline in the Anthropocene: death by a thousand cuts. *Proc. Natl Acad. Sci. USA* 118, e2023989118. 10.1073/pnas.2023989118 33431573 PMC7812858

[JEB252357C53] Waters, J. S. and Harrison, J. F. (2012). Insect metabolic rates. In *Metabolic Ecology: A Scaling Approach* (ed. J. Brown, R. Sibly and A. Brown), pp. 198-217. New York: John Wiley and Sons.

[JEB252357C54] Winston, M. L. (1992). *Killer bees: The African Honey Bee in the Americas*. Cambridge: Harvard University Press.

[JEB252357C55] Wohlgemuth, R. (1957). Die Temperaturregulation des Bienenvolkes unter regeltheoretischen Gesichtspunkten. *Z. Vgl. Physiol.* 40, 119-161. 10.1007/BF00297946

