# Supplementary material for: Limits and mechanisms of honey bee colonial thermoregulation in the heat
Source: J Exp Biol. 2026 Jul 23;229(14):jeb252357. doi: 10.1242/jeb.252357 (PMC13450896; doi:10.1242/jeb.252357)
Supplement: Supplementary file 1 [file jexbio-229-252357-s1.pdf]

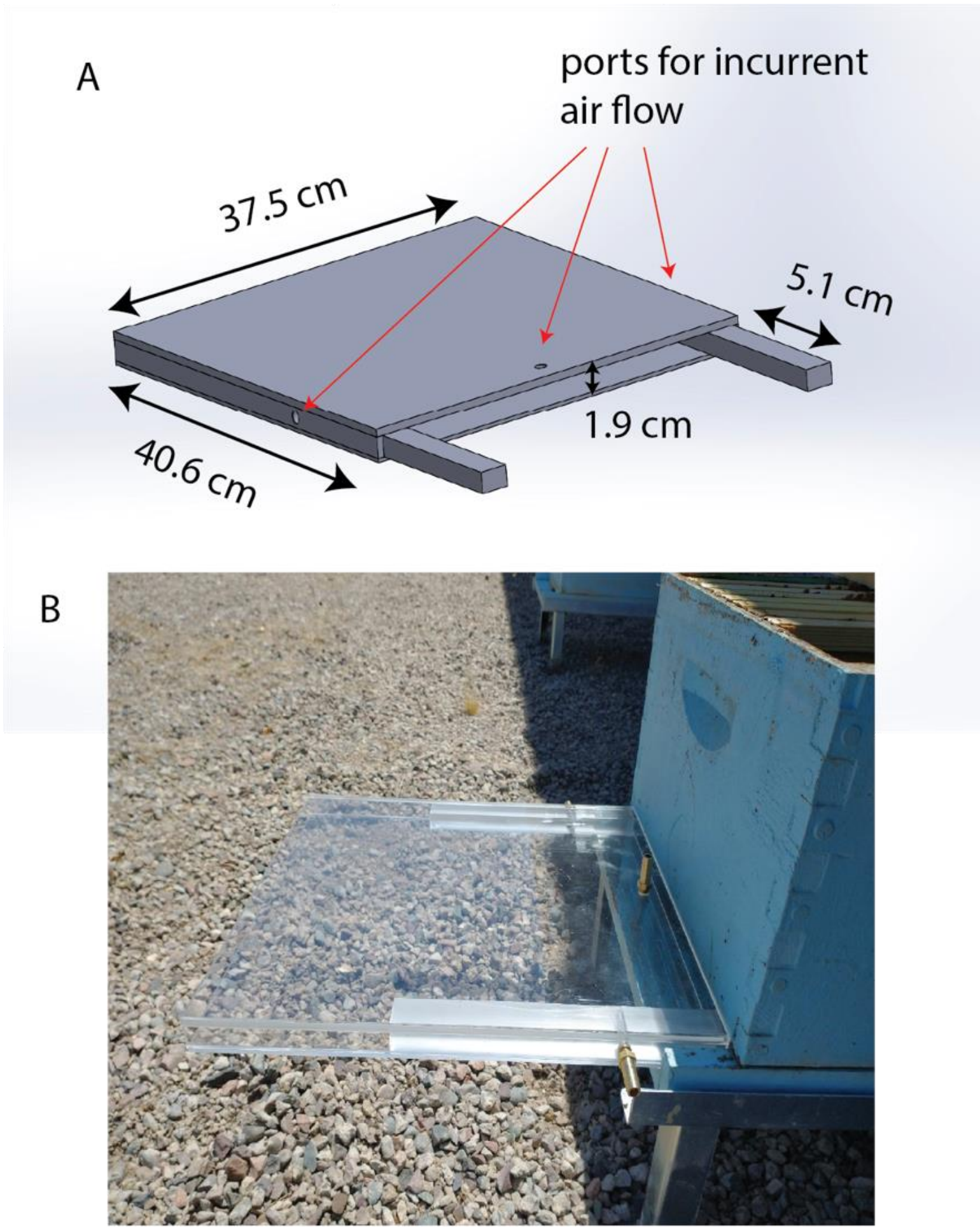

Fig. S1. Respiratory mask for a honey bee hive. A. Schematic with dimensions.

B. Photograph of mask inserted into hive entrance.

Journal of Experimental Biology • Supplementary information

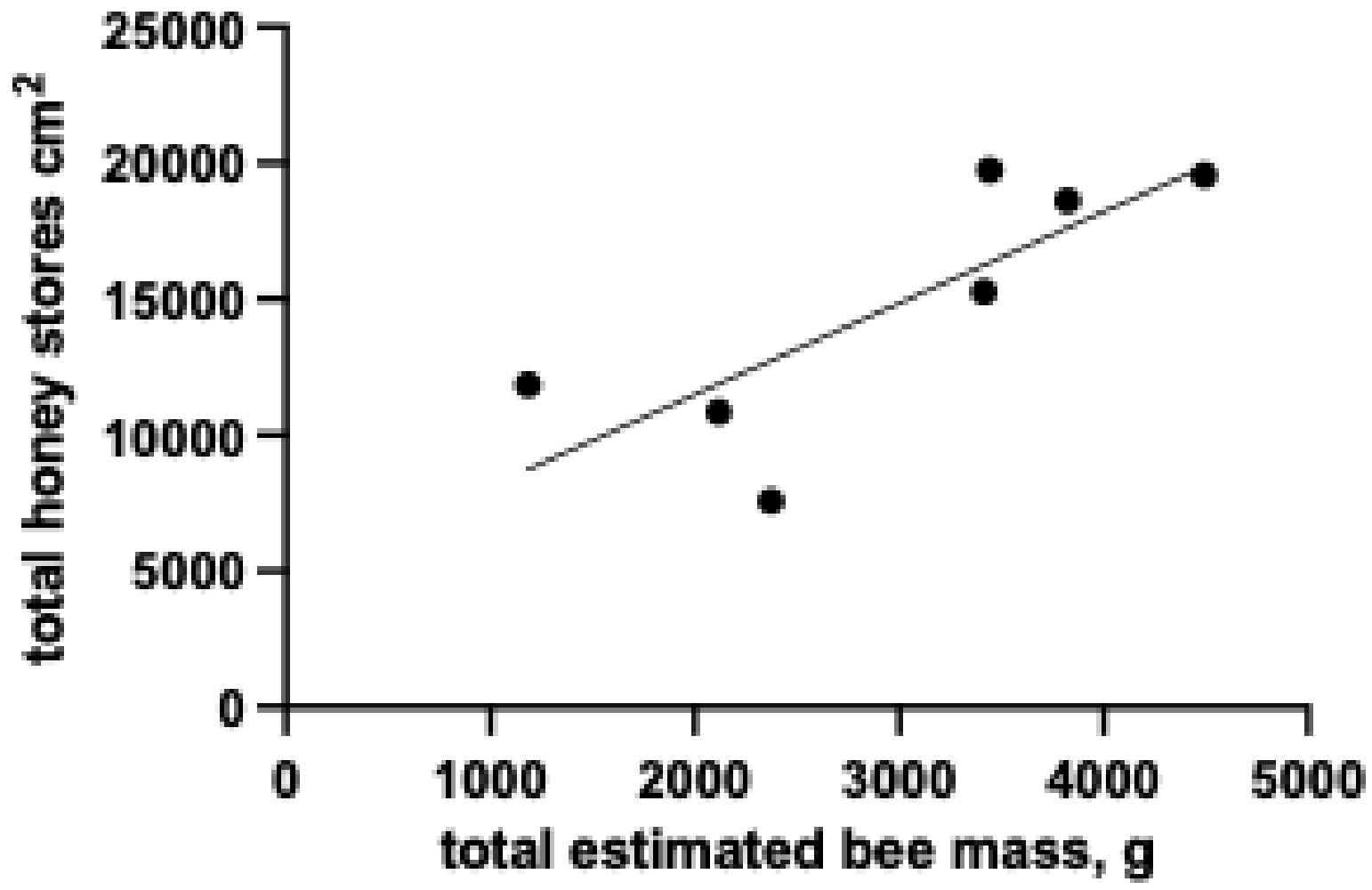

Fig. S2. Correlation (r = 0.79, r^2 = 0.63, P = 0.033) between total honey stores and estimated total bee mass for 2024 colonies. Using linear regression, total honey stores can be predicted as 4762 + 3.36 * total estimated bee mass.

.

Journal of Experimental Biology • Supplementary information

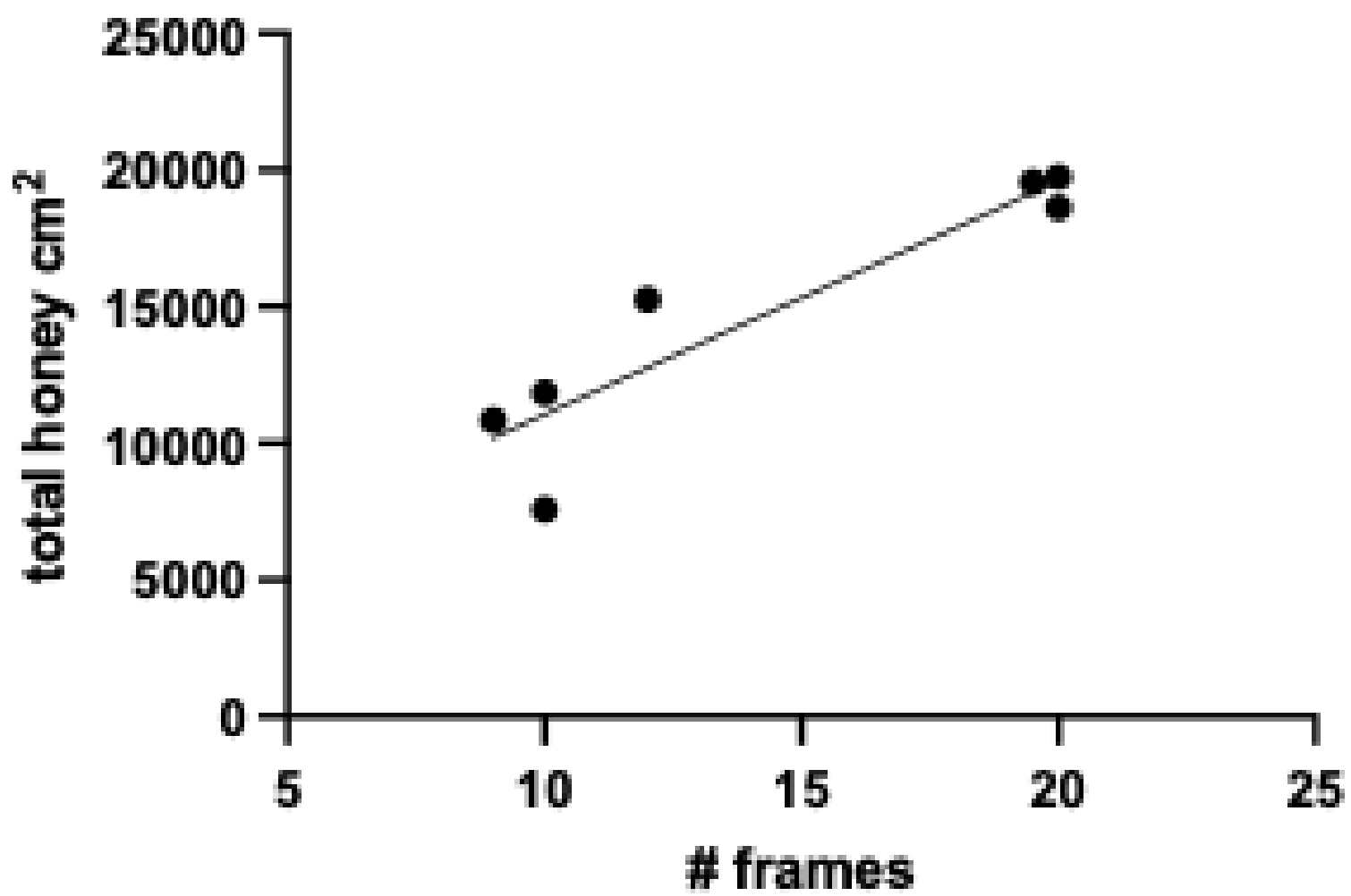

Fig. S3.. Correlation (r = 0.92, r^2 = 0.85, P = 0.003) between total honey stores and the number of occupied frames in the hive for 2024 colonies. Using linear regression, total honey stores can be predicted as 2542 + 852 * the number of occupied frames.

Journal of Experimental Biology • Supplementary information

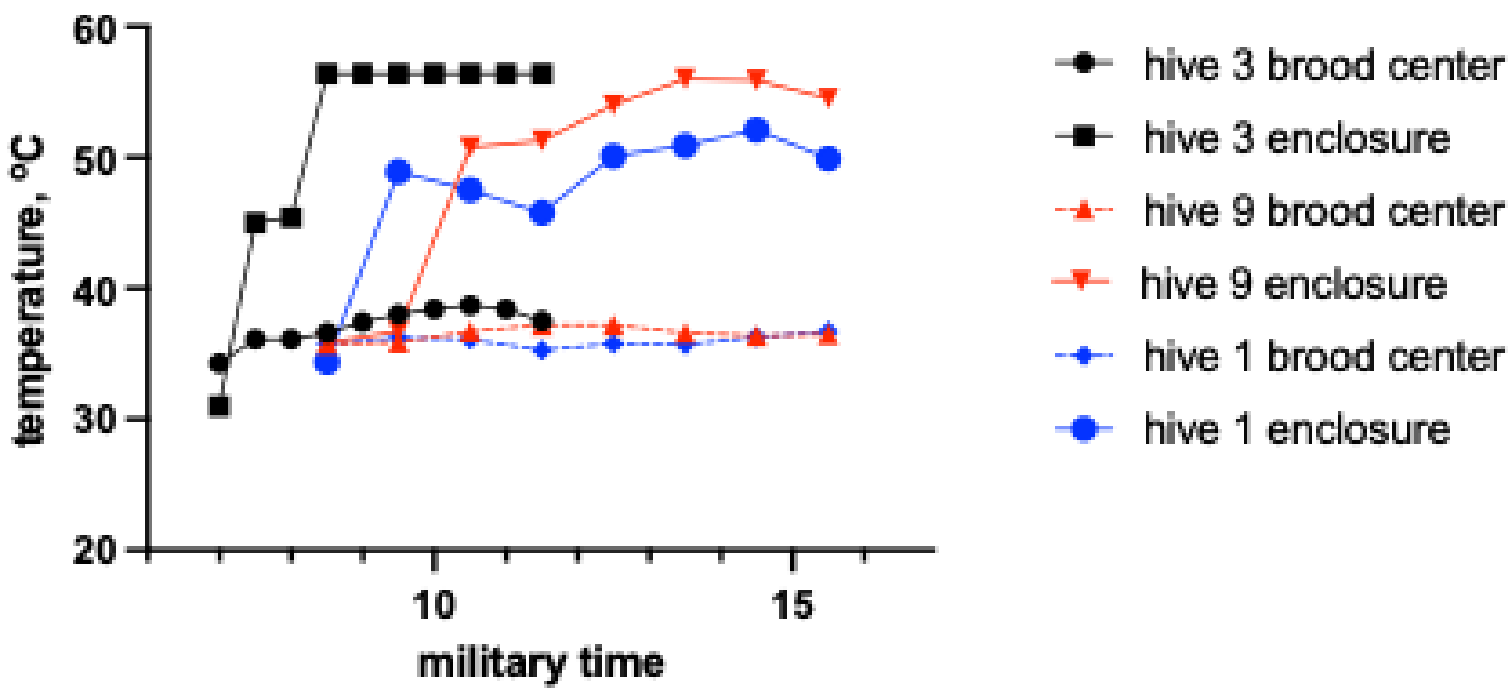

Fig. S4. Enclosure and brood center temperatures over time for three honey bee colonies exposed to >50ºC air temperature. For hive 3, brood center temperature rose steadily, whereas for hive 9 and 1, brood center temperatures rose, but then were relatively constant over time.

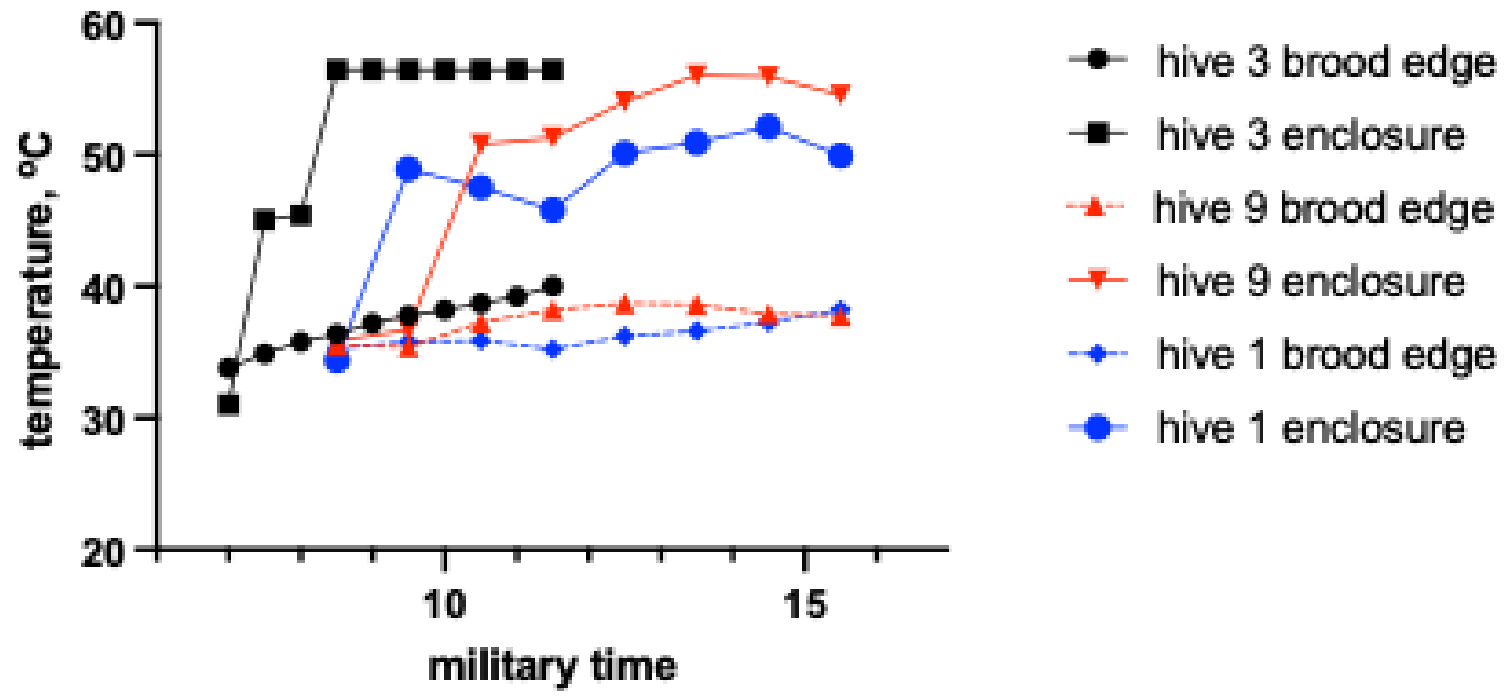

Fig. S5. Enclosure and brood edge temperatures over time for three honey bee colonies exposed to >50ºC air temperature. For all three colonies, brood edge temperatures rose relatively steadily over time.

Journal of Experimental Biology • Supplementary information

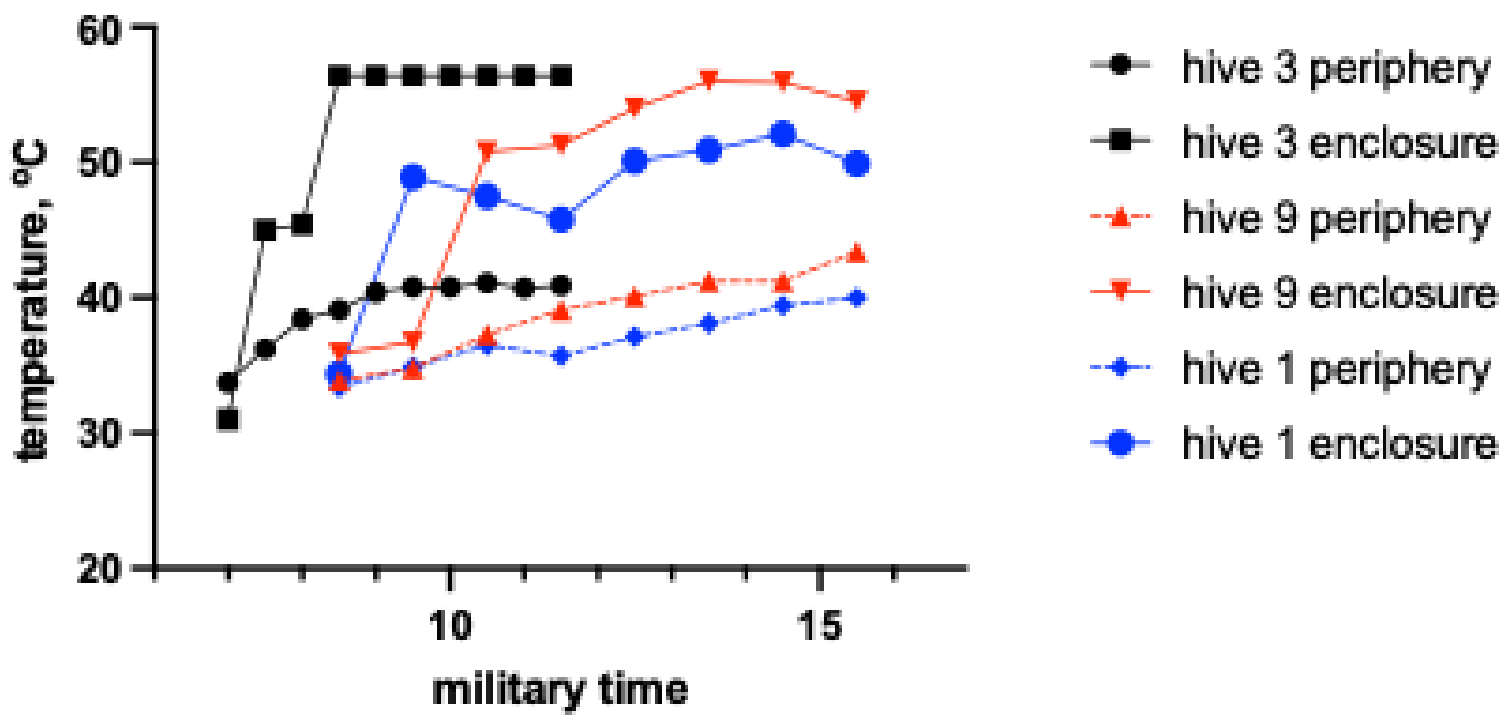

Fig. S6. Enclosure and hive periphery temperatures over time for three honey bee colonies exposed to >50ºC air temperature. For colony 3, hive periphery temperatures stabilized, but for colonies 9 and 1, periphery temperatures rose steadily over time.

Table S1. Sensitivity of estimated heat-buffering contributions to alternative parameter assumptions. Values indicate estimated rates of heat buffering during the experimental heat stress. Baseline values are from the primary heat-budget estimates in Fig. 6B. The bee redistribution scenario assumes that movement of adult bees toward the hive periphery approximately doubles estimated passive heat storage in bee bodies. The honey minus 50% scenario evaluates whether reducing estimated honey heat storage by half changes the qualitative ranking of thermoregulatory mechanisms. The combined scenario applies both assumptions simultaneously. Because passive heat-storage estimates scale linearly with mass, temperature change, and specific heat, changes in specific heat would alter the corresponding passive storage estimates proportionally. Across these scenarios, passive heat storage remains the dominant whole-colony heat-buffering mechanism, and honey remains one of the largest heat stores. Metabolic suppression and evaporative cooling are expressed as positive contributions to thermal buffering, representing reduced metabolic heat production and increased evaporative heat loss, respectively.

Scenario	Honey	Wax	Bees	Reduced metabolic heat production	Increased evaporative heat loss	Main conclusion
Baseline	13.3 W	4.0 W	3.2 W	1.6 W	0.6 W	Honey dominates; passive storage dominates
Bee redistribution	13.3 W	4.0 W	~6.4 W	1.6 W	0.6 W	Bees exceed wax, but not honey
Honey minus 50%	6.7 W	4.0 W	3.2 W	1.6 W	0.6 W	Passive storage dominates
Honey minus 50% + bee redistribution	6.7 W	4.0 W	~6.4 W	1.6 W	0.6 W	Honey/bees are similar; passive storage dominates

Journal of Experimental Biology • Supplementary information

Dataset 1. All data used for creation of the graphs and statistical analyses in this manuscript are available in this separate Excel file. For each hive, there are two rows, the initial (before heating) measurements, and the final measurements (after heating increased brood temperatures to 38 ºC). Included columns are the times for the initial and final measurements, total heating time, rates of colonial oxygen uptake, carbon dioxide emission and water loss in liters per minute, respiratory quotient (RQ), temperatures (ºC) recorded by loggers on top of the hive (enclosure temperature), entrance, brood center, brood edge, and the outside edge of the lower box, the number of incoming nonpollen foragers counted in five minutes, the number of pollen foragers counted in five minutes, the number of fanners observed in a scan sample, and whether bearding behavior was observed. Also recorded are the number of adults (this was measured before and after the heating trial if substantial bearding occurred), the number of larvae, number of pupae, and masses (grams) of adults, larvae, pupae, and all of these life stages summed. For the 2024 colonies and one 2022 colony, we recorded the number of frames with honey and the number of hive boxes. For the 2024 colonies, we measured the square cm of capped honey, uncapped honey, pollen, melted wax, and total honey (capped + uncapped).

Available for download at
https://journals.biologists.com/jeb/article-lookup/doi/10.1242/jeb.252357#supplementary-data

Dataset 2. This file shows the calculations used for estimating the contributions to thermoregulation during the heat stress. Row 4 provides the calculations and values for the active physiological mechanisms of thermoregulation (reduction in metabolic heat production and increase in evaporative heat loss). Rows 5-8 provide the sources of values used in row 4.

Row 12 provides the calculations and values for passive heat buffering in bees during the heat stress. Rows 13-26 provide references and calculations for the values used in row 4.

Available for download at
https://journals.biologists.com/jeb/article-lookup/doi/10.1242/jeb.252357#supplementary-data

Dataset 3. This file shows the calculation of median absolute differences for all of the variables.

Available for download at
https://journals.biologists.com/jeb/article-lookup/doi/10.1242/jeb.252357#supplementary-data
